# Evaluation of New Tools for Malaria Vector Control in Cameroon: Focus on Long Lasting Insecticidal Nets

**DOI:** 10.1371/journal.pone.0074929

**Published:** 2013-09-23

**Authors:** Josiane Etang, Philippe Nwane, Michael Piameu, Blaise Manga, Daniel Souop, Parfait Awono-Ambene

**Affiliations:** 1 Laboratory of Medical Entomology, Organisation de Coordination pour la lutte contre les Endémies en Afrique Centrale (OCEAC), Yaoundé, Cameroon; 2 Faculty of Medicine and Pharmaceutical Sciences, The University of Douala, Douala, Cameroon; 3 Faculty of Science, The University of Yaoundé I, Yaoundé, Cameroon; 4 Centre Supérieur des Sciences de la Santé, Université catholique d'Afrique Centrale, Yaoundé, Cameroun; 5 Division of Health Promotion, Ministry of Public Health, Yaoundé, Cameroon; 6 Service of Pesticide regulation, Ministry of Agriculture and rural development, Yaoundé, Cameroon; Tulane University School of Public Health and Tropical Medicine, United States of America

## Abstract

**Background:**

From 2006 to 2011, biological activity of insecticides for Indoor Residual Spraying (IRS), conventional treatment of nets (CTNs) or long lasting insecticidal nets (LLINs) was evaluated before their approval in Cameroon. The objective of the study was to select the best tools for universal malaria vector control coverage.

**Methodology:**

Bioassays were performed using WHO cones and the Kisumu susceptible strain of *Anopheles gambiae* s.s.. Among tested products, residual activity and wash resistance of Alpha-cypermethrin LLINs (Interceptor) and CTNs (Fendona) were assessed during 5 months in the Ntougou neighborhood.

**Principal Findings:**

All the 14 tested products were found effective (95–100% knockdown and mortality rates), although a significant decrease of efficacy was seen with lambda-cyhalothrinWP IRS, alpha-cypermethrin CTNs and LLINs (p< 0.05). However, the efficacy of Interceptor nets did not decrease during the 5 months evaluation, even after 25 washes (0.07<p< 0.9). Meanwhile Fendona SC nets displayed a drastic decrease of activity after 5 washes, odds ratio was 3.07 (1.0–8.59).

**Conclusion:**

This study provided useful data for decision making and community education toward universal coverage of malaria vector control in Cameroon.

## Introduction

The renewed effort to control malaria worldwide and move towards elimination is founded on the latest generation of effective tools and methods for prevention and treatment [1]. Vector control primarily through the use of insecticide indoor residual spraying (IRS) and long lasting insecticidal nets (LLINs) is the central pillar of this effort. According to WHO [2], LLINs should be considered a public good for populations living in malaria-endemic areas. Distribution of LLINs should be systematically accompanied by provision of information on how to hang, use and maintain them properly. In most settings where IRS has been or is being deployed, ITNs/LLINs are already in use and vise versa. It is believed that neither LLINs nor IRS alone will be sufficiently effective to achieve and maintain interruption of transmission in holo-endemic or hyper-endemic areas, especially in areas where vectors are developing resistance to insecticides. In 2004, WHO adopted integrated vector management (IVM) as the desired strategic approach for ecologically sound, cost-effective and sustainable control of vector-borne diseases [3], [4].

Indeed, several tools are being developed: longer-lasting IRS, non-pyrethroid LLINs, combined LLINs, pyrimiphos-methyl capsule suspension (CS) and chlofenapyr suspension concentrate (SC) for IRS, durable wall lining to complement IRS. New paradigms include spatial repellents, area-wide treatments, traps and targets, and Animal treatments [5]. However, strong scientific evidences are needed before these tools and strategies are recommended for wide scale implementation. Four LLINs are currently recommended by World Health Organization Pesticide Scheme (WHOPES) (Interceptor®, Yorkool®, Olyset®, PermaNet 2.0®); interim recommendation has been given for 9 additional LLNs as they are still under evaluation. Also in development are long-lasting treatment kits such as ICON® MAXX, designed to transform untreated nets into LLINs (as per the WHO definition) by simple dipping. Once such kits are approved, their use as an interim strategy to treat millions of untreated nets currently in use would have significant operational implications for rapidly increasing treatment coverage rates.

In Cameroon, malaria vector control using LLINs is highly prioritized. To move towards universal coverage, a national campaign was launched in August 2011 and two types of LLINs (Olyset®, 2% permethrin, Sumitomo Chemical CO., Japan; and PermaNet® 2.0 Vestergaard-Frandsen, Denmark) were used for free distribution. In addition, the reintroduction of IRS is being planned for the next coming years, while untreated nets bought from the local market by households may also need insecticide formulations for long-lasting treatment. However, field testing of new tools for vector control has been limited. Meanwhile, WHO recommends that National Programmes monitor and evaluate the performance of LLINs, under local conditions following procedures recommended in WHO guidelines to select the most suitable LLINs for their setting [6], [7]. The current study was carried out to determine initial and residual biological activity of some IRS and LLINs products in local conditions of use in Cameroon, as well as wash resistance of LLNs.

## Methods

### Study design

The current study was built in to prospective field and laboratory trials carried out during 5 years period from December 2006 to October 2011, based on the 2005 World Health Organization (WHO) Guidelines for Laboratory and Field Testing of IRS, ITNs and LLINs [8] with slight modifications based on other field trials [9],[10]. IRS formulations, ITNs or LLINs prior registration by national authorities were trialed in the laboratory and sometimes at the community level. The target population included residents of the Ntougou II quarter in Yaounde, the capital city of Cameroon. The description of this study site is given elsewhere [11].

Originally, the study aimed at evaluating the initial biological activity of the products before community use. However, alpha-cypermethrin coated nets were chosen as a case study to monitor their residual activity in normal conditions of use and washing at community level. Households who agreed to partake in this part of the study were randomized to receive either Interceptor ® LLIN or a similar conventional net treated with the same chemical (alpha-cypermethrin) for comparison. For IRS products, data on residual activity of 3/7 tested formulations were already published [11].

### Nets and insecticides

A total of 14 insecticide products were tested, among which 7 formulations for IRS and 7 ITNs (Table 1).There were two categories of ITNs: 2 conventionally treated nets (CTNs) and 5 LLINs. The CTN treatment kits used for this study were designed to transform untreated nets into LLINs. Insecticide products were gathered by the National Registration Board in Cameroon and sent to the OCEAC Laboratory for evaluation.

**Table 1:** Record of insecticide formulations and long lasting insecticidal nets tested between 2006 and 2011.

Trade name	Insecticide compound &Formulation	Dosage (g a.i/m^2^)	Class of insecticide	Method of use	Year of evaluation
FICAM® VC	Bendiocarb 80WP	0.4	C	IRS	2009
AGROBEN®	Bendiocarb 80WDP	0.12	C	IRS	2011
ANTOUKA®	Pyrimiphos-methyl 50 EC	1.0 g	OP	IRS	2011
K-Othrine®	Deltamethrin 250 WG	0.02	PY	IRS	2009
FENDONA®	Alpha-cypermethrin 50 WP	5% (50 ml/m^2^)	PY	IRS	2006
ICON®	Lambda-cyhalothrin 10 CS	0.025	PY	IRS	2009
BOXER®	Lambda-cyhalothrin 10 WP	0.05	PY	IRS	2010
Permanet® 2	Deltamethrin Coated	0.055	PY	LLIN	2008
Permanet®3	Deltamethrin Coated + Synergist	0.085 –0.115	PY	LLIN	2008
Life Net®	Deltamethrin Incorporated	0.34	PY	LLIN	2011
Olyset Net®	Permethrin Incorporated	2%	PY	LLIN	2011
INTERCEPTOR®	Alpha-cypermethrin Incorporated	0.2	PY	LLIN	2008
FENDONA®	Alpha-cypermethrin 6 SC CTN	0.027.7	PY	LLIN	2008
ICONET® Maxx	Lambda-Cyhalothrin10 CS CTN+ Binder	0.050	PY	LLIN	2008

(1) CS: capsule suspension; EC  =  emulsifiable concentrate; SC  =  suspension concentrate; WG  =  water dispersible granule;

WP  =  wettable powder; WDP =  wettable dispersible powder.

(2) OC =  Organochlorines; OP =  Organophosphates; C =  Carbamates; PY =  Pyrethroids.

(3) g a.i/m^2^ : grams of active ingredient per meter square.

(4) CTN: Conventional treated net; LLIN; long lasting insecticidal net; PY: pyrethroid; OP: organophosphate; C: carbamates; IRS: indoor residual spraying.

All the 7 formulations of insecticides tested for IRS were approved by WHOPES [12]. Among the 2 formulations for long lasting net treatment, only one product has been approved by the WHOPES (ICON®MAXX) although as interim [13]. Among the 5 LLINs, three have been fully approved (Interceptor®, Olyset®and PermaNet® 2.0) while the two others are interim (Permanet® 3.0, LifeNet®) [6].

The 14 tested products were made with 6 active ingredients (Table 1), e.g. one carbamate (Bendiocarb), one organoposphate (pyrimiphos-methyl) and 4 pyrethroids (pemethrin, deltamethrin, alpha-cypermethrin and lambda-cyhalothrin). Bendiocarb and pyrimiphos-methyl were aimed at IRS, while permethrin was incorporated in LLINs. Lambda-cyhalothrin was used for two types of treatments: IRS and long lasting treatment; while deltamethrin was used for IRS and coated LLINs. Only alpha-cypermethrin was used for the three types of treatments: IRS, long lasting treatment or incorporated LLINs.

For long lasting treatments with alpha-cypermethrin, nets were treated by the study team with sachets of alpha-cypermethrin (Fendona®) provided by the BASF Chemical Company, to obtain an operational dosage of 27.7 mg/m^2^. The treatment of the conventional nets took place at the Ntougou II neighborhood by a trained team of malaria prevention workers from OCEAC. All nets were identified with a unique number and allocated to households.

### Bioassays

Two replicates of each net type were tested, for a total of 14 nets. A 30×30 cm sample was cut from each net and subjected to a baseline bioassay. For IRS products, 2 rooms made of concrete walls were used for each insecticide formulation, for a total of 14 rooms.

The bio efficacy of insecticide treatments was evaluated by means of the Kisumu susceptible strain of *Anopheles gambiae*s.s. reared in laboratory conditions as described by Etang et al. [11]. Bioassays were carried out according to WHO protocol [14], using three to five days old non blood-fed female mosquitoes provided from the OCEAC insectary. For testing IRS, five WHO cones were fixed firmly on walls. Five mosquitoes were introduced in each cone by using a plastic aspirator. After 30 minutes exposure to treated wall, mosquitoes were transferred in white plastic labeled cups covered with untreated netting, and the knockdown rate was recorded 60 minutes post exposure (KD60). Then, mosquitoes were kept in the insectary and supplied with 10% sugar solution. Mortality rates were recorded after 24 hours holding period. Ten batches of five mosquitoes were used for each room, and 10 batches were exposed to control room sprayed with tap water. Bioassays were carried out at 25 ± 2°C temperature and 70–80% RH humidity. The same protocol was applied to assess the bio efficacy of mosquito nets, except that the period of exposure was 3 minutes. Batches of ten mosquitoes were exposed to untreated netting as control.

### Wash resistance of alpha-cypermethrinLLINs

Net samples from alpha-cypermethrin LLINs and CTNs were washed after baseline bioassays and subject to other bioassays 6 days after each washing. Two washing procedures were used: the first one in the Laboratory using a protocol adapted from the standard WHO washing procedure for phase I and the second one in the community using their traditional procedures for washing clothes.

In the laboratory, net samples were washed at weekly intervals, by placing a single sample in a 1–l Erlenmeyer flash with soap solution. The soap solution was made by thoroughly mixing 0.5 g of soap (le chat®, savon de Marseille, France) with 250 ml of water. The net and soap solution were shaken for 10 minutes on a shaker bath [8] at 155 rotations per minutes at 30°C. After washing, the nets were rinsed two times by shaking for 10 minutes in 250 ml of distilled water and then hung to dry for 24 h.

At the community level, nets were washed every 2 weeks by mothers of the households using their usual dress washing procedures, in 15 liter container with 5liters of tap water. However, the study team recommended them to use the local and common mild household soap (CCC® namely Cameroon Chemical Company®). The entire net was soaked in water, then rubbed with soap, rinsed twice and hung on a washing line for an hour to dry.

### Data analysis

Knockdown and mortality rates were calculated and analyzed according to WHO criteria [14] to determine whether IRS, ITNs/LLINs were effective. Treatment was considered effective when KD60 and mortality rates in exposed mosquitoes were >98%; mortality rates between 98% and >80% or Knockdown rates between 98 and 95% indicate a decrease of treatment efficacy. Below this threshold, the treatment was considered as non effective. A two level ordered logit model was used to study variations of LLINs efficacy over time of use or after washing. Two models were involved; the first with the variable determined from KD60 rates and the second determined from mortality rates. We looked at the effects of covariates on the odds of the probability that a net is effective against the probability of reduced efficacy or not effective. The likelihood ratio test (LR) was used to compare the efficacy of nets after different “types of washing”, e.g. Laboratory versus Community washing. All the analyses were performed using the R function *sabre* in the R package *sabreR* built in the R 2.15.0 software.

### Informed consent and ethical approval

All heads of households were informed about the study prior to initiation. Quarter leaders helped create awareness of the study within the community. The head of each household was asked to sign the consent form for their household to participate in this evaluation. Community members were informed that participation in the study was completely voluntary and that they may withdraw from the study at any time without penalty. This study was approved by the Ministry of Health Review Board in Cameroon.

## Results

### Control Assays

No knockdown effect was observed after exposure of mosquitoes to control walls or nets. The mortality rates recorded was always below 5%. No side effect of IRS, ITNs or LLINs was reported by inhabitants.

### Bio efficacy of IRS insecticide formulations

**Figure 1:**
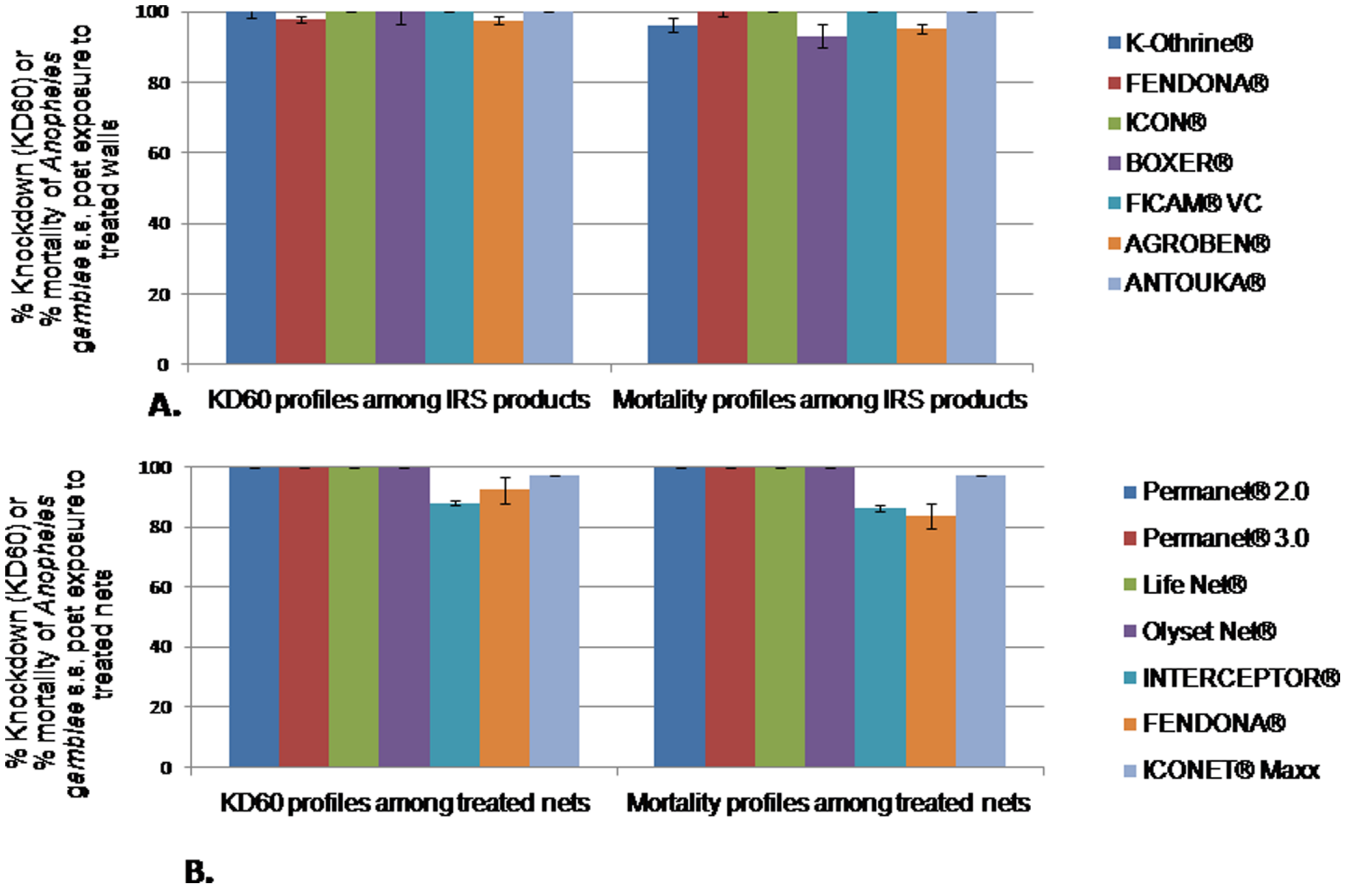
Knockdown and mortality rates of susceptible *Anopheles gambiae* s.s. post contact with different insecticide treatments. A: Indoor Residual Spraying; B: Long lasting insecticidal nets; KD60: knockdown rate of mosquitoes 60 minutes post contact to treated material. All tested IRS insecticide formulations were found effective although a decrease of insecticide activity was seen with lambda cyhalothrin 10 WP (P<0.05). All tested insecticidal nets were found effective although a decrease of insecticide activity was seen with alpha cypermethrin conventional or long lasting treated nets (P<0.05).

For IRS insecticide formulations, bio efficacy was evaluated one week after wall treatments. Overall, most of the tested insecticide formulations were found effective against *An. gambiae* s.s. The knockdown and mortality rates were mostly 98–100% (Figure 1A); except for the lambda-cyhalothrin 10 WP which displayed a slight but significant decrease of mortality rates (93%, p< 0.05), suggesting a low level bio efficacy.

The residual activity of 3 products (Bendiocarb WP, Lambda-cyhalothrin CS and deltamethrin WG) was monitored among the 7 IRS formulations tested; these data are available elsewhere [Etang et al., 2011].

### Bioefficacy of ITNs and LLINs

Most of the tested insecticide treated nets were found effective against *An. gambiae* s.s. The KD60 and mortality rates were very high (98–100%), except the alpha-cypermethrin CTNs (Fendona SC treated nets) and LLINs (Interceptor nets) which displayed less than 95% KD60 and 83–87% mortality rates ( p< 0.05) (Figure 1B), suggesting a low level bio efficacy. No significant difference was seen between Interceptor and Fendona SC unused nets either in term of KD60 or mortality rates (p = 0.903).

### Residual bio efficacy of alpha-cypermethrin LLINs

**Figure 2:**
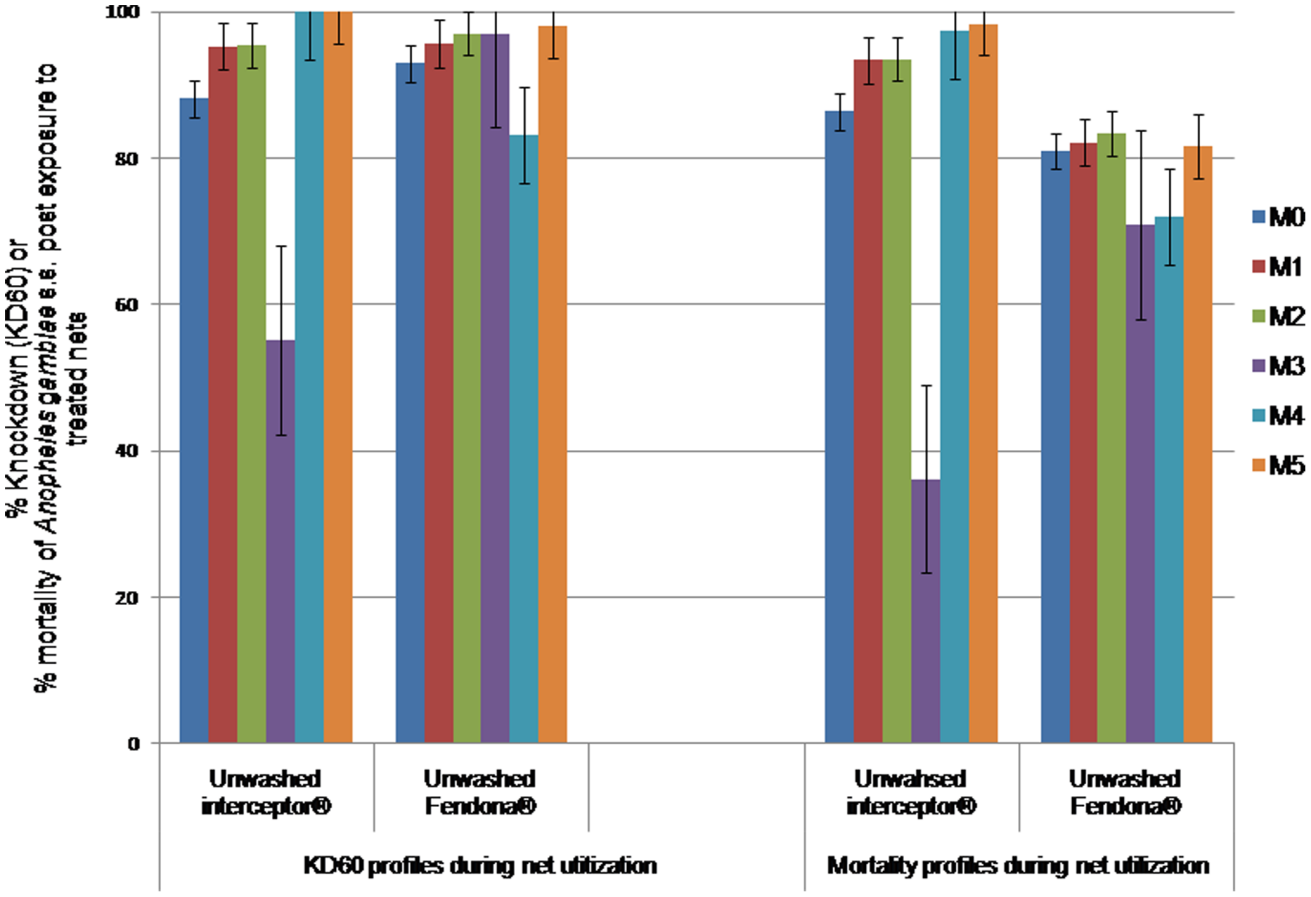
Knockdown and mortality rates of susceptible *Anopheles gambiae* s.s. post contact with community used Alpha-cypermethrin long lasting nets. M0, M1, M2, M3, M4, M5: month 0, month 1, month 2, month 3, month 4, month 5; KD60: knockdown rate of mosquitoes 60 minutes post contact to treated material. KD60 and mortality rates for interceptor nets remained very during the 5 month trial except on month 3when tested nets were found very dirty. With Fendona SC long lasting treated nets, the KD60 and the mortality rates were slightly but significantly lower than those recorded with Interceptor nets (p<0.05).

With Interceptor nets, the KD60 rates significantly increased from 88–96% during the first 2 months of evaluation to 100% during months 4 and 5 (p<0.009). The mortality rates also increased from 86–94% during the first 2 months to 97–99% during months 4 and 5, but the difference was not significant (0.079<p<0.967). However, the KD60 and mortality rates were drastically decreased on the 3^rd^ month (55% and 36% respectively), suggesting a decline of net efficacy at that time (p<0.01) (Figure 2, Table 2). With Fendona SC long lasting treated nets, the KD60 and the mortality rates during the 5 months of net utilization were around 83–98% and 70–83% respectively. The difference between Interceptor and Fendona SC nets was not significant in term of KD60 rates (p = 0.851), while the mortality rates with Fendona SC nets was significantly lower than those recorded with Interceptor nets (p = 0.032, Table 2). The lowest mortality rates (70–72%) were recorded during the 3^rd^ and 4^th^ months of Fendona SC nets utilization, e.g. below the WHO threshold of efficacy.

**Table 2:** Two level proportional odds regression models for the efficacy of LLINs determined from kd60 (model 1) and mortality (model 2).

	Coefficient		Stand. error		Z-value		P-value	
Variable	*Model 1*	*Model 2*	*Model 1*	*Model 2*	*Model 1*	*Model 2*	*Model 1*	*Model 2*
**Type of washing**								
Laboratory	–37.406	–36.812	0.618	0.863	–60.493	–42.677	0.000	0.000
Field	–36.855	–37.263	0.498	0.758	–74.027	–49.179	0.000	0.000
Unwashed	–35.930	–35.738	0.431	0.548	–83.309	–65.255	0.000	0.000
**Type of net (Ref = Fendona)**								
Interceptor	–0.047	1.124	0.388	0.524	–0.122	2.145	0.903	0.032
**Number of washing**	–0.007	–0.113	0.035	0.053	–0.188	–2.146	0.851	0.032
Kd60	0.515	0.0711	0.003	0.005	151.31	15.160	0.000	0.000
Mortality	0.018	0.467	0.004	0.005	4.692	93.038	0.000	0.000
**Time (Ref = M0)**								
M1	0.223	–0.718	0.720	1.052	0.310	–0.683	0.757	0.495
M2	1.125	–1.163	0.552	0.871	2.039	–1.336	0.041	0.182
M3	1.765	–0.042	0.672	1.027	2.626	–0.041	0.009	0.967
M4	2.429	1.421	0.616	0.809	3.945	1.758	0.000	0.079
M5	2.835	–0.568	0.687	0.857	4.127	–0.663	0.000	0.507
**Cut points**								
Cut 1	14.955	7.608	0.344	0.537	43.416	14.176	0.000	0.000
Cut 2	16.940	16.961	0.357	0.610	47.430	27.797	0.000	0.000
**Net level random effect variance**	0.25E-15	0.11E-15	0.329	0.369	0.76E-15	0.30E-15	1	1
**Number of observations**				56				

The random effect variances at the net level in both KD60 and mortality models were not significant (p = 1), suggesting that there was no unmeasured heterogeneity at the level of the net which was not captured by the covariates in the model (Table 2).

### Wash resistance of alpha-cypermethrin LLINs

**Figure 3:**
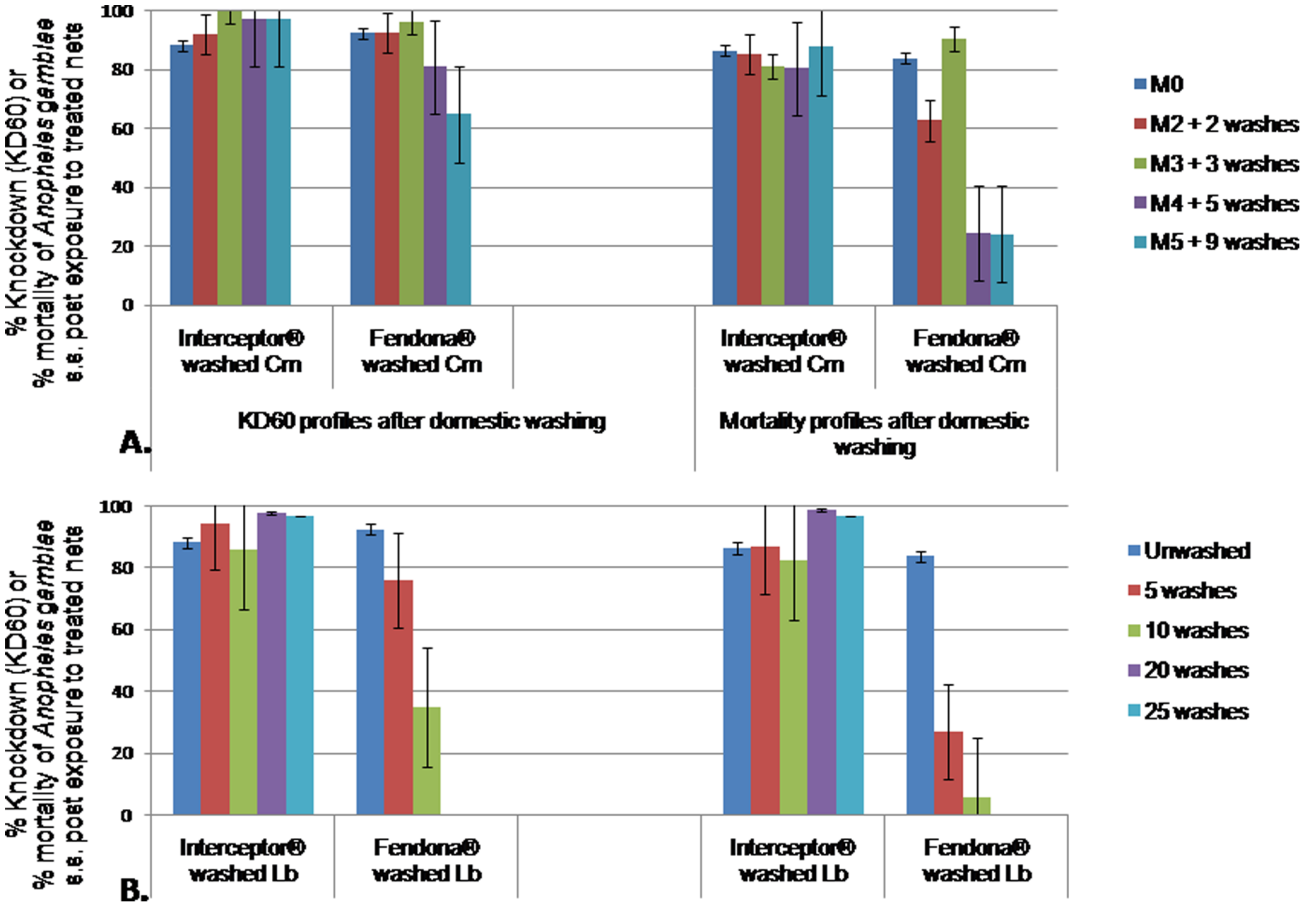
Knockdown and mortality rates of susceptible *Anopheles gambiae* s.s. post contact with Alpha-cypermethrin long lasting washed nets. A: Nets used and washed in community; B: Nets washed in laboratory conditions, M0, M1, M2, M3, M4, M5: month 0, month 1, month 2, month 3, month 4, month 5; KD60: knockdown rate of mosquitoes 60 minutes post contact to treated material; washed CM: nets washed in the community; washed Lb: nets washed in the laboratory. Interceptor Nets remained highly effective after washing up to 25 times in the laboratory or 9 times at community level, although a slight but significant decrease of KD60 and mortality rates was seen after each washing cycle (p<0.05). With Fendona, the KD60 and mortality rates remained high until 3washes either in the lab or at community level. No significant difference of wash resistance was seen between laboratory and community washing procedures (p<0.05).

A total of 9 washes were carried out at community level (Figure 3A) and up to 25 washes in laboratory conditions (Figure 3B).

With Interceptor nets washed either at the community level or in laboratory conditions, no significant decrease of KD60 or mortality rates was observed, no matter the number of washes (p = 0.851), although some fluctuations were observed from one series of washing to another. The KD60 rates varied from 86% to 100%, while the mortality rates varied between 80% and 88%.

Conversely, the Fendona treated nets efficacy dropped progressively from each series of washing to another especially after the 5^th^ washing series either in laboratory conditions or at community level (75–81% KD60 and 24–26% mortality rates) (p<0.032). Nevertheless, Fendona SC washed nets were found more effective than unwashed ones during the third month of utilization when they were found very dirty (P<0.05).

No significant difference was seen in bio-efficacy of nets washed at community level compared with those washed in laboratory conditions, either with Interceptor or Fendona nets (LR = 0.217, p =  0.897 for KD60 rates and LR  =  0.0147, p = 0.993 for mortality rates), suggesting that washing procedures at community level was well done.

## Discussion

Increasing use of LLINs and IRS provides an unprecedented opportunity to control and, in some countries, eliminate malaria [1], [2], [15], [16], [17]. The 14 tested products were made with 6 active ingredients among which 4 pyrethroid insecticides. From the point of view of both safety and effectiveness, pyrethroids are the best insecticides ever developed for public health use. They account for the majority of IRS coverage worldwide and are the only class of insecticides recommended in ITNs and LLINs. The reliance of modern malaria control on pyrethroids and the increasing resistance of malaria vectors to these products put current global efforts at risk. It is therefore crucial to emphasize on non pyrethroid insecticides IRS products that are recommended by WHO. Accordingly, three formulations made of non-pyrethroids (one organophosphate and two carbamates) were tested as alternative products for malaria vector control in Cameroon.

Data from this study underline several aspects of operational research including evaluation of new formulations for IRS, new concepts of LLINs as well as awareness for education of the community on good nets’ handling attitudes. The 14 tested products were found relatively effective against *An*. *gambiae* s.s.; this level of efficacy is consistent with the WHO full or interim recommendations for their wide scale implementation. Indeed, they are expected to be effective in protecting against malaria provided that people use them properly and consistently, although the residual bio efficacy is to be confirmed for many of these products. Among the 7 LLINs/CTNs tested in this study, alpha cypermethrin long lasting CTN and LLINs were slightly less effective. This result may be related to a high level of exito-repellent and irritancy effects of alpha cypermethrin formulations used for net treatments, compared with other tested pyrethroid formulations. Exito-repellent and irritancy effects are known to reduce mosquito contact with treated nets, resulting in the decrease of their mortality rates, especially with new nets prior to their utilization [18]. Accordingly, the KD60 and mortality rates reported in the current study were significantly increased following reduction of exito-repellent and irritancy effects by domestic use and washing process.

WHOPES supervised laboratory and field trials have demonstrated wash resistance and efficacy of Interceptor nets [19].Various field trials have shown further evidence that Interceptor nets are effective against malaria vectors in different countries or settings [20], [21], [22], [23], [24]. WHOPES published interim recommendations for Interceptor in 2006 based on phase I laboratory testing and phase II experimental hut studies. Full WHO recommendations have been given in 2012. In the current study, the follow up of the net’s bio efficacy at community level revealed an increase of knockdown and mortality rates during their utilization. Despite similarities in material strength, the Interceptor® LLINs was found to significantly outperform CTNs, these results are consistent with previous studies [22]. In India, Interceptor® was reported to contain an average of 126 mg/m^2^ of residual insecticide after 12 months of use, which is above the level normally associated with effective vector control when using alpha-cypermethrin (40 mg/m^2^) in conventional treatment [25].

However in the current study, Interceptor nets were found less effective during the 3^rd^ in which they were very dirty. The impact of dirt and fume on the efficacy of ITNS has already been investigated by several authors, highlighting the importance of good net washing process to ensure its continued insecticidal effect. Erlanger et al. [26] reported that the effective usable life of rarely or non retreated nets was closer to 2–3 years. After that time nets are dirty, hardly with any insecticide and full of holes. The mean number of washes was estimated at 8.6 times per year. Nevertheless, it seems that nets still provide some protection even if torn, dirty and without insecticide [27]. Although LLINs are designed to resist repeated washing, excessive or aggressive washing and the use of harsh detergents (such as some traditional soap) may rapidly reduce their useful life. A wide range of variables such as the number of times a bed net was washed, how it was washed, UV exposure, handling and wear of the bed net, exposure to dust and soot, and other variables may affect insecticide retention and residual bio efficacy.

Beyond research that focused on washing nets in a laboratory or in tightly controlled field setting, the current study also examined how well LLINs performed in a real-world field setting, with no control or supervision over the behavior of the villagers using and washing the bed nets. Net users were just asked to wash nets with mild soaps and not with detergent. Indeed, Interceptor LLNs was found to retain effective bio-efficacy causing >98% mortality in *An. gambiae* s.s. and withstood 25 laboratory washes. The community compliance and acceptance was high and no side effects were reported during the entire course of the study. The users required better awareness about the upkeep of nets and washing practices. Bhatt et al. [28] estimated the duration of an effective intervention based on Interceptor LLN to be of about three years in central India. The frequency of washing of nets by their users ranged from one wash per year to 24 washes per year, and more than 80% of LLNs were found washed at community level [28]. As many as13 brands of local soaps and soap powders were used by the villagers for washing the nets. This may also be the case in Cameroon. The reason for more frequent washes of nets is related with dirt from house sweeping and soot from jungle frequency of net washing by their users ranged from one wash per year to 24 washes per year wood burning for cooking. The dirty looks given by the net is considered to be unhealthy and socially unacceptable as it has been reported in studies carried out in urban Dar-es-Salaam [29].

Contrarily to Interceptor, the resistance of long lasting alpha-cypermethrin CTN was not more than three washes. These data are similar to those reported by Gimning et al [30] on a rapid decrease of knockdown and mortality rates, using the kisumu *An. gambiae* s.s. strain in contact with some deltamethrin and permethrin candidate long lasting insecticidal nets after 3 washing in laboratory conditions. A significant correlation between number of washes and residual deltamethrin concentration was reported by Norris et Norris [31] in Zambia, with a subsequent decrease of residual bio efficacy against *An*. *gambiae* s.s.

## Conclusions

In addition to recent data on residual bio efficacy of some insecticide formulations for IRS on different types of walls, current data provide baseline information to guide the planning of Integrated Vector Management for malaria control in Cameroon. The 14 insecticide products were found relatively effective against a susceptible colony of *An. gambiae* s.s., the major malaria vector in Cameroon. Furthermore, the highest rate of washing of nets by their users or at laboratory was estimated at 25 washes and 3 washes for Interceptor and Fendona respectively. It is critical to seize this opportunity and rapidly expand access to these new technologies for all populations at risk of malaria. To increase current ITN coverage, the untreated or conventionally treated nets currently in use should be treated using long lasting treatment kits, once such kits are available. In addition, proper LLINs care should be known as strong determinant of LLINs efficacy; hence education on the importance of their use and care is key when distributing LLINs.
